# Nucleation of superfluid-light domains in a quenched dynamics

**DOI:** 10.1038/s41598-018-30789-9

**Published:** 2018-08-24

**Authors:** Joaquín Figueroa, José Rogan, Juan Alejandro Valdivia, Miguel Kiwi, Guillermo Romero, Felipe Torres

**Affiliations:** 1 Departamento de Física, Facultad de Ciencias, Universidad de Chile, Casilla 653, Santiago, 7800024 Chile; 2 Center for the Development of Nanoscience and Nanotechnology 9170124, Estación Central, Santiago, Chile; 3 Departamento de Física, Universidad de Santiago de Chile (USACH), Avenida Ecuador 3493, 9170124 Santiago, Chile

## Abstract

Strong correlation effects emerge from light-matter interactions in coupled resonator arrays, such as the Mott-insulator to superfluid phase transition of atom-photon excitations. We demonstrate that the quenched dynamics of a finite-sized complex array of coupled resonators induces a first-order like phase transition. The latter is accompanied by domain nucleation that can be used to manipulate the photonic transport properties of the simulated superfluid phase; this in turn leads to an empirical scaling law. This universal behavior emerges from the light-matter interaction and the topology of the array. The validity of our results over a wide range of complex architectures might lead to a promising device for use in scaled quantum simulations.

## Introduction

The absence of energy dissipation in the flow dynamics of a quantum fluid is one of the most fascinating effects of strongly correlated condensates^1–6^. Quantum phase transitions, from Mott insulator to superfluid, have been observed in a wide range of physical platforms such as ultracold atoms in optical lattices^7^, trapped gases of interacting fermionic atom pairs^8^, and exciton-polariton condensates^9–11^. Furthermore, the remarkable progress in controlling light-matter interactions in the microwave regime of circuit quantum electrodynamics (QED) has provided a suitable scenario for studying strongly correlated effects with light^12–14^. In this case, coupled resonator arrays (CRAs) each doped with a two-level system (TLS) allow for the formation of dressed quantum states (polaritonic states) and effective photon-photon interactions. The underlying physics is well described by the Jaynes-Cummings-Hubbard (JCH) model^15–17^. In this case, if the frequencies of the single resonator mode and the TLS are close to resonance, the effective photonic repulsion prevents the presence of more than one polaritonic excitation in the resonator, due to the photon-blockade effect^18–20^. Detuning the atomic and photonic frequencies diminishes this effect and leads the system to a photonic superfluid^16^. Unlike Bose-Einstein condensation in optical lattices, polariton condensation includes two kind of excitations, atomic and photonic, and the transition from Mott-insulator to superfluid is accompanied by a transition of the excitations from polaritonic to photonic^16^.

Here we show how a first-order like phase transition of the simulated superfluid phase of polaritons in CRAs can be induced by a quench dynamics as described by the JCH model. We compare full numerical simulations of several arrangements of CRAs with mean-field theory of photonic fluctuations dynamics. In this case, the simulated Mott-superfluid transition relies on the topological properties of the array, since the on-site photon blockade strongly depends on the connectivity of each node, even for small resonator-resonator hopping strength. When the system is prepared in the Mott state with a filling factor of one net excitation per site, and a sudden quench of the detuning between the single resonator mode and the TLS is applied, we find a first-order like phase transition which can be described by two bosonic excitations of the lower and upper polariton band. We find that a nucleated superfluid photon state emerges in a localized way, which depends on the topology of the array. This avalanche-like behavior near the simulated phase transition leads to a universal scaling law between critical parameters of the superfluid phase and the average connectivity of the array.

## The Model

**Figure 1:**
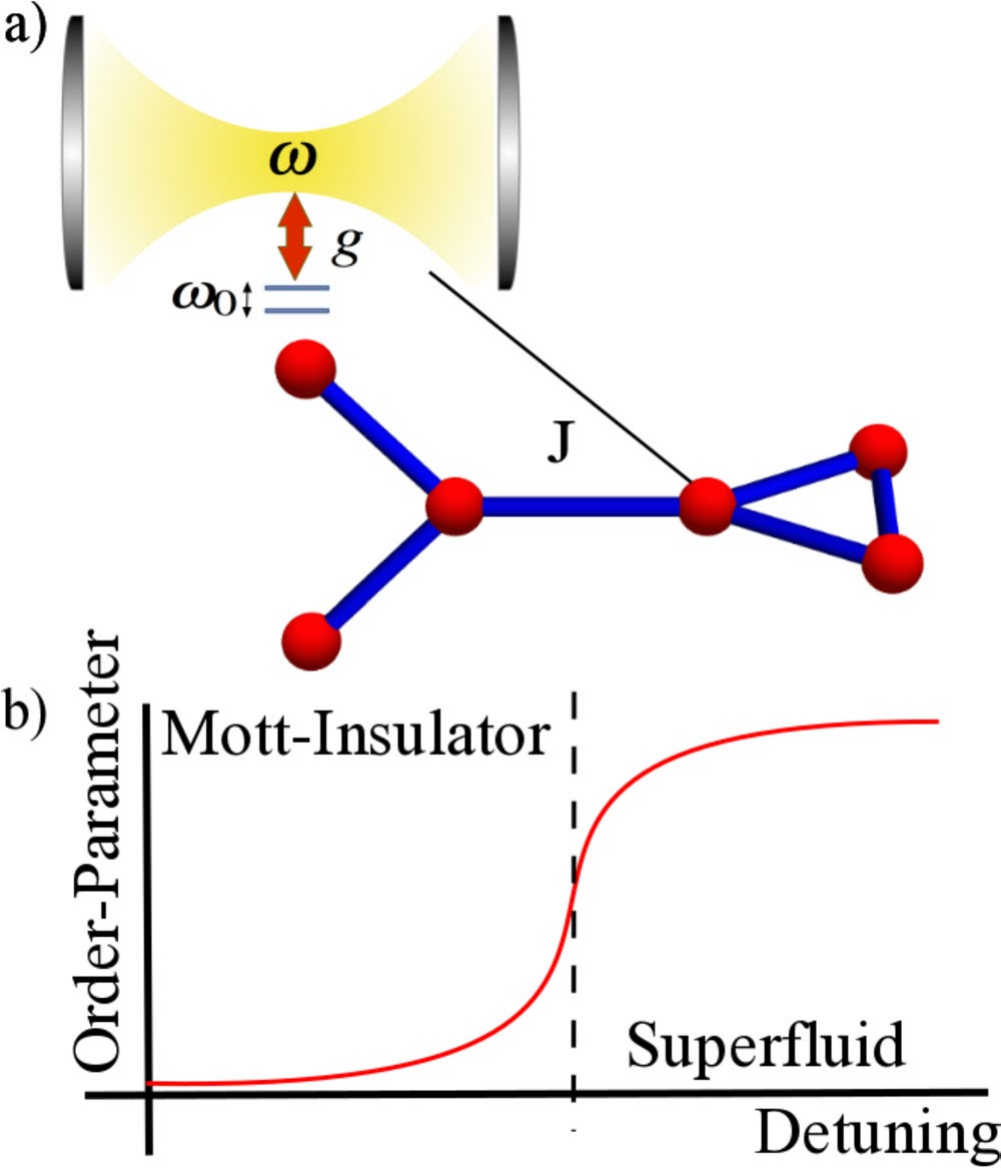
(**a**) Schematic representation of the Jaynes-Cummings-Hubbard lattice in a complex array where each node consists of a single resonator strongly coupled to a two-level system. (**b**) Phase transition from Mott-insulator to superfluid in light-matter CRAs systems as a function of the detuning parameter.

The physical scenario that we consider are CRAs in complex arrangements such as the one in Fig. 1(a). Here, each node of the array consists of a QED resonator doped with a TLS to be a real or artificial atom, and the whole system is described by the Jaynes-Cummings-Hubbard model^15–17^, whose Hamiltonian reads

1 $${H}_{{\rm{JCH}}}=\sum _{i\mathrm{=1}}^{L}\,{H}_{i}^{{\rm{JC}}}-J\sum _{\langle i,j\rangle }\,{A}_{ij}{a}_{i}^{\dagger }{a}_{j}+{\rm{h}}\mathrm{.}{\rm{c}}\mathrm{.}-\sum _{i\mathrm{=1}}^{L}\,{\mu }_{i}{n}_{i},$$

where *L* is the number of lattice sites, ${a}_{i}({a}_{i}^{\dagger })$ is the annihilation (creation) bosonic operator, *J* is the photon-photon hopping amplitude, *A*_*ij*_ is the adjacency matrix which takes values *A*_*ij*_ = 1 if two sites of the lattice are connected and *A*_*ij*_ = 0 otherwise. *μ*_*i*_ stands for the chemical potential at site *i* and ${n}_{i}={a}_{i}^{\dagger }{a}_{i}+{\sigma }_{i}^{+}{\sigma }_{i}^{-}$ represents the number of polaritonic excitations at site *i*. Also, ${H}_{i}^{{\rm{JC}}}=\omega {a}_{i}^{\dagger }{a}_{i}+{\omega }_{0}{\sigma }_{i}^{+}{\sigma }_{i}^{-}+g({\sigma }_{i}^{+}{a}_{i}+{\sigma }_{i}^{-}{a}_{i}^{\dagger })$ is the Jaynes-Cummings (JC) Hamiltonian describing light-matter interaction^21^. Here, ${\sigma }_{i}^{+}({\sigma }_{i}^{-})$ is the raising (lowering) operator acting on the TLS Hilbert space, and *ω*, *ω*_0_, and *g* are the resonator frequency, TLS frequency, and light-matter coupling strength, respectively. Notice that the total number of elementary excitations (polaritons) in this system $N={\sum }_{i}^{M}({a}_{i}^{\dagger }{a}_{i}+{\sigma }_{i}^{+}{\sigma }_{i}^{-})$ is the conserved quantity [*N*,*H*_*JCH*_] = 0^22,23^.

The quantum dynamics of this model has been studied for linear lattices^15,16^, and its equilibrium properties at zero temperature have been studied by means of density matrix renormalization group^24^, and by means of mean field (MF) theory, for two-dimensional lattices^17,25,26^ and complex networks^27^. The latter studies have provided evidence of a quantum phase transition from Mott-insulating phases to a superfluid polaritonic phase. Beyond the MF approach there have been important contributions from the numerical and analytical viewpoint for extracting the phase boundaries^28–31^, the study of critical behavior^30,31^, and the excitation spectrum^29–31^. For a general overview on many-body physics with light relevant literature is available^32–34^.

## Mott-insulator to superfluid phase transition

Here we briefly summarize the Mott-insulator to superfluid phase transition in the JCH model^16^. Our main results are focused on the quantum dynamics of the JCH model (1) in complex networks, where we focus on the canonical ensemble with a fixed total number of polaritons^13,14^. In this case, the JCH Hamiltonian reads

2 $${H}_{{\rm{JCH}}}=\sum _{i=1}^{L}\,{H}_{i}^{{\rm{JC}}}-J\sum _{\langle i,j\rangle }\,{A}_{ij}{a}_{i}^{\dagger }{a}_{j}+{\rm{h}}\mathrm{.}{\rm{c}}\mathrm{.}$$

In the atomic limit, where the photon-hopping can be neglected ($J\ll g$), the JC Hamiltonian at site *i* (${H}_{i}^{{\rm{JC}}}$) can be diagonalized in the polaritonic basis that mixes atomic and photonic excitations |*n*, ±〉_*i*_ = *γ*_*n*±_|↓, *n*〉_*i*_ + *ρ*_*n*±_|↑,*n* − 1〉_*i*_ with energies ${\varepsilon }_{n}^{\pm }=n\omega +{\rm{\Delta }}\mathrm{/2}\pm \chi (n)$, where $\chi (n)=\sqrt{{{\rm{\Delta }}}^{2}\mathrm{/4}+{g}^{2}n}$, *ρ*_*n*+_ = cos(*θ*_*n*_/2), *γ*_*n*+_ = sin(*θ*_*n*_/2), *ρ*_*n*−_ = −*γ*_*n*+_, *γ*_*n*−_ = *ρ*_*n*+_, $\tan \,{\theta }_{n}=2g\sqrt{n}/{\rm{\Delta }}$, and the detuning parameter Δ = *ω*_0_ − *ω*.

Now, one can introduce the polaritonic creation operators at site *i* defined as ${P}_{i}^{\dagger (n,\alpha )}=|n,\alpha {\rangle }_{i}\langle \mathrm{0,}-|$, where *α* = ± and we identify |0,−〉≡|↓, 0〉 and |0, + 〉≡|$\rlap{/}{0}$〉 being a ket with all entries equal to zero, that is, it represents an unphysical state. These identifications imply *γ*_0−_ = 1 and *γ*_0 +_ = *ρ*_0±_ = 0. Using this polaritonic mapping the Hamiltonian (2) can be rewritten as^16,26^

3 $$H=\sum _{i=1}^{L}\sum _{n=1}^{\infty }\sum _{\alpha =\pm }{\varepsilon }_{n}^{\alpha }{P}_{i}^{\dagger (n,\alpha )}{P}_{i}^{(n,\alpha )}-J\sum _{\langle i,j\rangle }\,{A}_{ij}[\sum _{n,m=1}^{\infty }\sum _{\alpha ,\alpha ^{\prime} ,\beta ,\beta ^{\prime} }\,{t}_{\alpha ,\alpha ^{\prime} }^{n}{t}_{\beta ,\beta ^{\prime} }^{m}{P}_{i}^{\dagger (n-\mathrm{1,}\alpha )}{P}_{i}^{(n,\alpha ^{\prime} )}{P}_{j}^{\dagger (m,\beta )}{P}_{j}^{(m-\mathrm{1,}\beta ^{\prime} )}+{\rm{h}}\mathrm{.}{\rm{c}}\mathrm{.}],$$

where ${t}_{\pm +}^{n}=\sqrt{n}{\gamma }_{n\pm }{\gamma }_{(n-\mathrm{1)}+}+\sqrt{n-1}{\rho }_{n\pm }{\gamma }_{(n-\mathrm{1)}-}$ and ${t}_{\pm -}^{n}=\sqrt{n}{\gamma }_{n\pm }{\rho }_{(n-\mathrm{1)}+}+\sqrt{n-1}{\rho }_{n\pm }{\rho }_{(n-\mathrm{1)}-}$. The first term in Eq. (3) stands for the local polaritonic energy with an anharmonic spectrum and gives rise to an effective on-site polaritonic repulsion. The last term in Eq. (3) represents the polariton hopping between nearest neighbors and long range sites, and it may also allow for the interchange of polaritonic excitations.

If the physical parameters of the Hamiltonian (3) are in the regime $Jn\ll g\sqrt{n}\ll \omega$, and for an integer filling factor, where the total number of excitations *N* over the lattice is an integer multiple of the number of unit cells *L*, the lowest energy state is the product ${\otimes }_{i=1}^{L}\mathrm{|1,}\,-{\rangle }_{i}$ which corresponds to a Mott-insulating phase, and its associated energy is $E=N{\varepsilon }_{1}^{-}$. In the thermodynamic limit, the interplay between the on-site polariton repulsion and the polariton hopping leads to a phase transition from a Mott insulator to a superfluid phase. The latter may be reached by diminishing the on-site repulsion by means of detuning the atomic and photonic frequencies. At equilibrium, this phase transition may be quantified by means of bipartite fluctuations^24,35^. In a simulated Mott-insulator transition, where an adiabatic dynamics drives the passage, it has been shown that a suitable order parameter corresponds to the variance of the number of excitations per site. Figure 1(b) shows the archetypal behavior of the order parameter as a function of the detuning Δ in the adiabatic dynamic regime, and for an integer filling factor of one net excitation per site^16^.

## Quenched dynamics and Topology in finite-size complex lattices

Our aim is to describe how complex arrangements of CRAs, such as the one appearing in Fig. 1(a), affect the simulated phase transition from Mott insulator to superfluid as the detuning parameter Δ is suddenly quenched. In particular, we are interested in how one can manipulate photonic transport properties of the emerging superfluid phase depending on the specific topology of the CRAs. As order parameter we choose the time-averaged standard deviation of the polariton number $\frac{1}{T}{\int }_{0}^{T}\,dt{\sum }_{i}^{L}\,(\langle {n}_{i}^{2}\rangle -{\langle {n}_{i}\rangle }^{2}))$ with *T* = *J*^−1^, and we assume the whole system initially prepared in the Mott-insulating state $|{\psi }_{0}\rangle ={\otimes }_{i=1}^{L}\mathrm{|1,}\,-{\rangle }_{i}$, with Δ = 0 at each lattice site. In the Supplementary Material we present another equivalent measure of the order parameter based on the bipartite fluctuation proposed by S. Rachel *et al*.^35^, and D. Rossini *et al*.^24^. Of course, due to computational restrictions, we consider relatively small arrangements of CRAs, but with varying degrees of complexity, suggesting that the topology of the network could be used in a nontrivial way to manipulate the emerging of the superfluid phase as these system becomes larger and approach the thermodynamic limit. The initialization process may be achieved by the scheme proposed by Angelakis *et al*.^16^. For instance, in circuit QED^13,14^ one might cool down the whole system reaching temperatures around *T*_0_ ~ 15 mK. In this case, the system will be prepared in its global ground state $|G\rangle ={\otimes }_{i\mathrm{=1}}^{L}\mathrm{|0,}-{\rangle }_{i}$. Then, one can apply individual magnetic fields on the TLSs, each implemented via a transmon qubit^36^, such that the resonance condition Δ = 0 is achieved. This way one can address individually each cavity with an external AC microwave current or voltage tuned to the transition |↓, 0〉_*i*_→|1, −〉_*i*_, with a driving frequency *ω*_*D*_ = *ω* − *g*, such that the system will be prepared in the desired initial state |*ψ*_0_〉. The sudden quench of the detuning can be achieved by applying magnetic fields to the transmon qubits in order to reach the desired superfluid phase. It is noteworthy that when the initial state is a linear superposition of upper and lower polariton states (Δ ≠ 0) the quantum dynamics will be dominated by these two polaritonic bands. Also, we carry out full numerical calculations for the parameters *g* = 10^−2^*ω* and *J* = 10^−2^*g*, and we consider up to 6 Fock states per bosonic mode. These parameter values allow us to prevent the interchange of polaritonic excitations between different sites.

**Figure 2:**
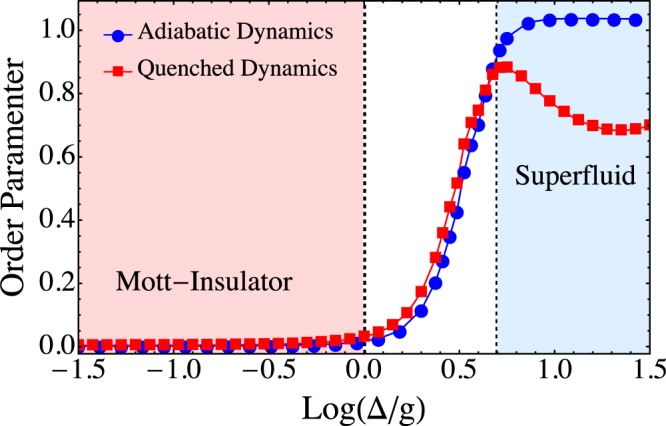
Quantum phase transition of a dimer array. Detuning dependence of the order parameter with two TLS coupled through photon hopping, adiabatic dynamics (blue circles) and quench dynamics (red squares). Continuous lines have been added as guide to the eye.

**Figure 3:**
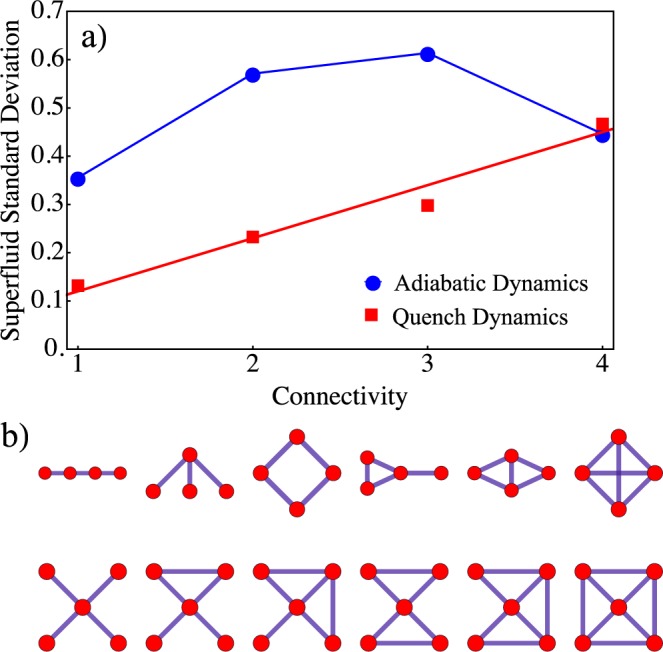
(**a**) Standard deviation of the superfluid phase as a function of the connectivity. Adiabatic dynamics (blue circles) and quench dynamics (red squares). A set of CRAs with four and five interconnecting resonators, as shown in (**b**) are considered. Continuous lines have been added as a guide to the eye.

In order to gain insight into the quench dynamics of the topological CRAs let us consider a dimer array. As shown in Fig. 2, the simulated Mott-insulator to Superfluid phase transition strongly depends on the type of dynamics. Adiabatic dynamics resembles a second order phase transition which leads to a continuous change of the state of the system. On the other hand, the quench dynamics takes place accompanied by a discontinuous change of the state, analogous to the Metal-Insulator transition of oxides^37^. Hence, as we expected, the adiabatic dynamics is not qualitatively affected by the distribution of nearest neighbors. However, the topological properties of the array dominate a first-order like phase transition driving the quench dynamics (see Fig. 2). As the degree of inter-connectivity between the resonators grows the distance between them rapidly diminishes, and thus local correlations become more important due to quantum interference effects. If scaled up to the size of the system, due to the increase in the degrees of freedom, the numerical simulation time grows exponentially. In the next section we obtain an empirical scaling law to address this issue. Indeed, we demonstrate that the photon propagation in the simulated superfluid phase strongly depends of the connectivity per site ${k}_{i}={\sum }_{j}\,{A}_{ij}$. Let us consider a set of arrays with a fixed number of TLS. As shown in Fig. 3(a) in the quench dynamics case the averaged standard deviation depends linearly on the connectivity, which means that depending on the connectivity the local superfluid states are reached with different detuning scales. We consider a set of CRAs with four and five interconnecting resonators as shown in Fig. 3(b). In contrast to these results, the adiabatic dynamics does not exhibit a monotone or linearly growing behavior, which leads to a sharper phase transition, as illustrated in Fig. 2.

## Mean-field theory of the Superfluid Phase

In the thermodynamic limit, the emergent superfluid phase behaves as a quantum liquid^17^. Superfluidity is achieved by means of a transition of the excitations from polaritonic to photonic. In order to describe the simulated superfluid phase in our system, we introduce the photonic order parameter^17^ *ψ* = 〈*a*_*i*_〉. Using the decoupling approximation ${a}_{i}^{\dagger }{a}_{j}\approx \langle {a}_{i}^{\dagger }\rangle {a}_{j}+{a}_{i}^{\dagger }\langle {a}_{j}\rangle -\langle {a}_{i}^{\dagger }\rangle \langle {a}_{j}\rangle$, the resulting mean-field JCH Hamiltonian can be written as

4 $${H}_{JCH}=\sum _{i}\,{H}_{i}^{JC}-J\sum _{i}\,{k}_{i}(\psi {a}_{i}^{\dagger }+{\psi }^{\ast }{a}_{i}\mathrm{).}$$

Therefore, the simulated Mott-insulator phase can be characterized by the on site repulsion, which suppresses the fluctuations of the number of per site excitations |*ψ*| = 0. On the contrary, the superfluid phase is dominated by the hopping and the quantum fluctuations |*ψ*| ≠ 0. Now we focus on the light-matter coupling induced by the hopping of photons through cavities. Introducing the identity *σ* ^+^ *σ*^−^ + *σ*^−^*σ* ^+^  = *I*, we obtain an effective light-matter coupling, since it retains the mixed products of photonic and two level operators,

5 $${h}_{i}^{LM}={\tilde{g}}_{i}{a}_{i}^{\dagger }{\sigma }_{i}^{-}+{\tilde{g}}_{i}^{\dagger }{a}_{i}{\sigma }_{i}^{+}+{\rm{h}}\mathrm{.}{\rm{c}}\mathrm{.}$$

**Figure 4:**
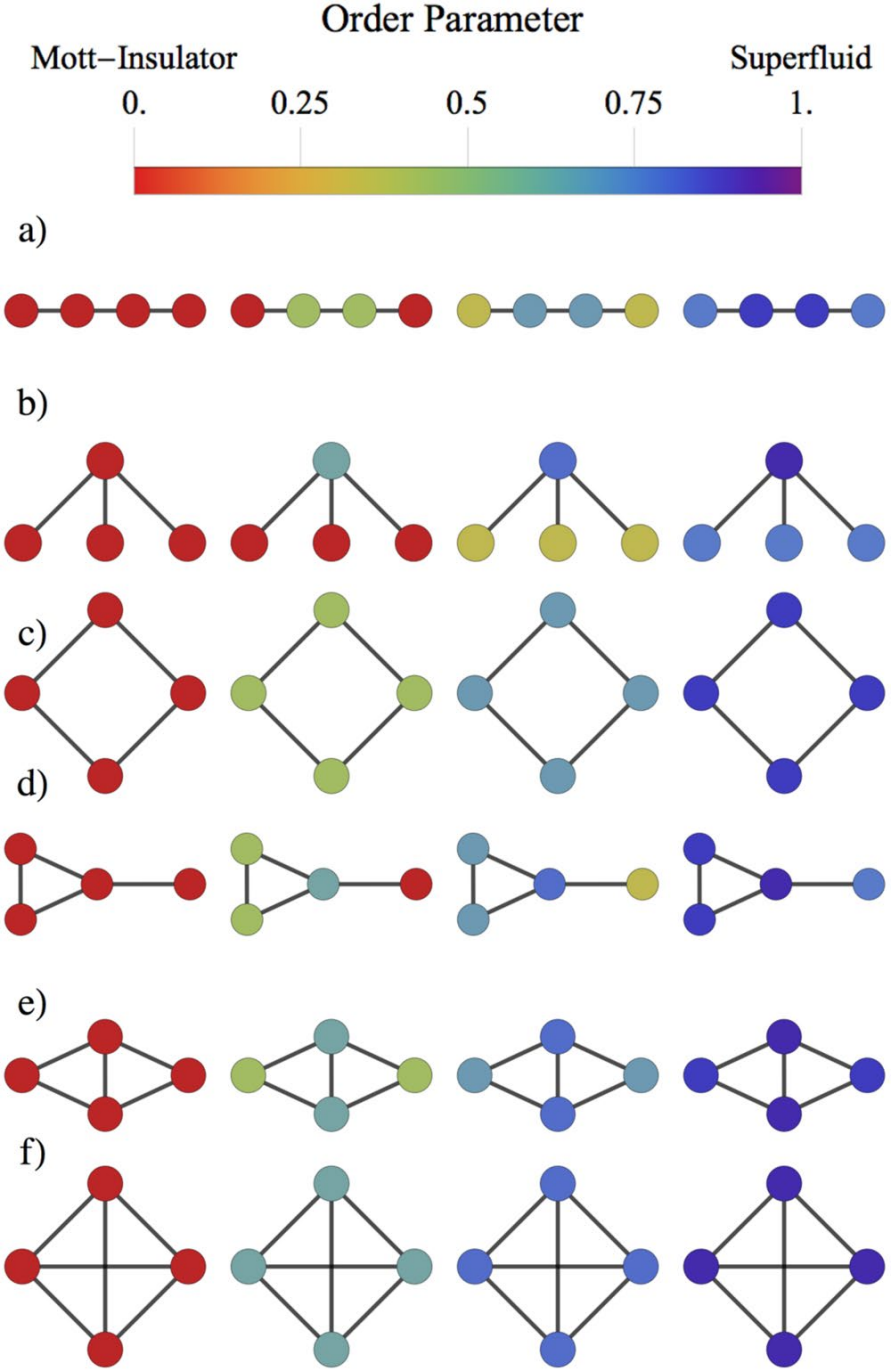
Numerical simulation of the quench dynamics. The full set of four node arrays, with (**a**,**b**) three; (**c**,**d**) four; (**e**) five; and (**f**) six connections. Connectivity per site (**a**) (1, 2, 2, 1), (**b**) (1, 1, 1, 3), (**c**) (2, 2, 2, 2), (**d**) (1, 3, 2, 2), (**e**) (2, 3, 3, 2), and (**f**) (3, 3, 3, 3). As the connectivity is increased locally the superfluid phase is achieved with a lower detuning strength. For each array and from left to right we have considered parameters *log*(Δ/*g*) = (0.5, 0.7, 0.75, 0.8), and *g* = 10^−2^*ω*, *J* = 10^−3^*ω*, where *ω* is the resonator frequency.

Here ${\tilde{g}}_{i}=Ig-J{k}_{i}\psi {\sigma }_{i}^{+}$ is the effective light-matter coupling per site, which therefore turns out to be an operator. In the simulated superfluid phase the atomic transitions are expected to be suppressed against the photonic dressed states. Moreover, the total excitation number does not change, hence when the photonic excitations increase the atomic excitations decrease. Note that when ${\tilde{g}}_{i}=Ig$, i.e. when there are no hopping or topological effects,

6 $$\langle {\sigma }_{i}^{+}\rangle =\frac{g}{J{k}_{i}}\frac{1}{\psi },$$

which indicates that the total number of excitations is conserved and also demonstrates that the increase of the photonic states leads to a reduction of the atomic excitations, due to the conservation of the number excitations. Figure 4 shows the effect of the quench dynamics on the simulated phase transition of the JCH model for different arrays. In this case the nucleation of superfluid states emerges due to the variation of the order parameter, according to Eq. (6). In the Mott-Insulator state $\langle {\sigma }_{i}^{+}\rangle  > 0\,\forall \,i$, when the detuning is increased $\langle {\sigma }_{i}^{+}\rangle$ decreases by a factor 1/(*k*_*i*_*ψ*), until the superfluid phase is reached.

We have shown that the mean field approach strongly supports the scaling law of the order parameter shown in Fig. 3(a); namely, as the connectivity of CRAs is increased locally, the light superfluid phase is achieved for a smaller detuning strength.

## Conclusion

We show that quench dynamics induce a first-order like phase transition in coupled resonator arrays doped with a two-level system. The nucleation of simulated superfluid states has been demonstrated by numerical simulation and by a mean field theoretical approach. In the quench dynamics the abrupt change of the order parameter, instead of sharper crossover driven by adiabatic dynamics, is explained by the non uniform transition from Mott-Insulator to superfluid, which locally depends of the connectivity. Since the quench dynamics exhibits the same behavior independent of the choice of the order parameter, the standard deviation of the polariton number or the bipartite fluctutation, our results reveal the universality of the simulated first order phase transition (also see Supplementary Material). As the number of TLS is increased the averaged standard deviation of the superfluid phase depends linearly on the connectivity. At an increased scale, for large networks of doped optical/microwave resonators, our system may enter the field of quantum simulators. In particular, as far as we understand, there is no known microscopic mechanism for predicting nucleation in first-order phase transitions. In this context, our results provide an exact geometrical description for the appearance of domain nucleation due to the number of connections. Thus, our results may be used to predict, and manipulate, the nucleation of a superfluid phase of light in complex-random networks.

## Electronic supplementary material

Supplementary Information

## References

[1] Kapitza P (1938). Viscosity of liquid helium below the *λ*-point. Nature.

[2] Leggett, A. J. *Quantum Liquids* (Oxford University Press, 2006).

[3] Anderson MH, Ensher JR, Matthews M, Wieman CE, Cornell EA (1995). Observation of bose-einstein condensation in a dilute atomic vapor. Science.

[4] Onofrio R (2000). Observation of superfluid flow in a bose-einstein condensed gas. Phys. Rev. Lett..

[5] Zwierlein MW (2003). e. a. Observation of bose-einstein condensation of molecules. Phys. Rev. Lett..

[6] Schiró M, Bordyuh M, Öztop B, Türeci HE (2012). Phase transition of light in cavity qed lattices. Phys. Rev. Lett..

[7] Greiner M, Mandel O, Esslinger T, Hansch TW, Bloch I (2002). Quantum phase transition from a superfluid to a mott insulator in a gas of ultracold atoms. Nature.

[8] Regal CA, Greiner M, Jin DS (2004). Observation of resonance condensation of fermionic atom pairs. Phys. Rev. Lett..

[9] Lerario G (2017). Room-temperature superfluidity in a polariton condensate. Nature Physics.

[10] Wertz E (2010). Spontaneous formation and optical manipulation of extended polariton condensates. Nature Physics.

[11] Byrnes Tim, Kim Na Young, Yamamoto Yoshihisa (2014). Exciton–polariton condensates. Nature Physics.

[12] Houck Andrew A., Türeci Hakan E., Koch Jens (2012). On-chip quantum simulation with superconducting circuits. Nature Physics.

[13] Raftery J, Sadri D, Schmidt S, Türeci HE, Houck AA (2014). Observation of a dissipation-induced classical to quantum transition. Phys. Rev. X.

[14] Fitzpatrick M, Sundaresan NM, Li ACY, Koch J, Houck AA (2017). Observation of a dissipative phase transition in a one-dimensional circuit qed lattice. Phys. Rev. X.

[15] Hartmann MJ, Brandão FGSL, Plenio MB (2006). Strongly interacting polaritons in coupled arrays of cavities. Nature Physics.

[16] Angelakis DG, Santos MF, Bose S (2007). Photon-blockade-induced mott transitions and *xy* spin models in coupled cavity arrays. Phys. Rev. A.

[17] Greentree AD, Tahan C, Cole JH, Hollenberg LCL (2006). Quantum phase transitions of light. Nature Physics.

[18] Birnbaum KM (2005). e. a. Photon blockade in an optical cavity with one trapped atom. Nature.

[19] Imamoḡlu A, Schmidt H, Woods G, Deutsch M (1997). Strongly interacting photons in a nonlinear cavity. Phys. Rev. Lett..

[20] Greentree AD, Vaccaro JA, R de Echaniz S, Durrant AV, Marangos JP (2000). Prospects for photon blockade in four-level systems in the n configuration with more than one atom. Journal of Optics B: Quantum and Semiclassical Optics.

[21] Jaynes ET, Cummings FW (1963). Comparison of quantum and semiclassical radiation theories with application to the beam maser. Proceedings of the IEEE.

[22] Hartmann M, Brandão F, Plenio M (2008). Quantum many-body phenomena in coupled cavity arrays. Laser & Photonics Reviews.

[23] Hartmann MJ, Plenio MB (2007). Strong photon nonlinearities and photonic mott insulators. Physical Review Letters.

[24] Rossini D, Fazio R, Santoro G (2008). Photon and polariton fluctuations in arrays of qed-cavities. EPL (Europhysics Letters).

[25] Na N, Utsunomiya S, Tian L, Yamamoto Y (2008). Strongly correlated polaritons in a two-dimensional array of photonic crystal microcavities. Phys. Rev. A.

[26] Koch J, Le Hur K (2009). Superfluid mott-insulator transition of light in the jaynes-cummings lattice. Phys. Rev. A.

[27] Halu A, Garnerone S, Vezzani A, Bianconi G (2013). Phase transition of light on complex quantum networks. Phys. Rev. E.

[28] Rossini, D. & Fazio, R. Mott-insulating and glassy phases of polaritons in 1d arrays of coupled cavities. *Phys. Rev. Lett*. **99**. 10.1103/PhysRevLett.99.186401 17995423

[29] Aichhorn M, Hohenadler M, Tahan C, Littlewood PB (2008). Quantum fluctuations, temperature, and detuning effects in solid-light systems. Phys. Rev. Lett..

[30] Pippan P, Evertz HG, Hohenadler M (2009). Excitation spectra of strongly correlated lattice bosons and polaritons. Phys. Rev. A.

[31] Schmidt S, Blatter G (2009). Strong coupling theory for the jaynes-cummings-hubbard model. Phys. Rev. Lett..

[32] Hartmann MJ (2016). Quantum simulation with interacting photons. Journal of Optics.

[33] Noh C, Angelakis DG (2016). Quantum simulations and many-body physics with light. Rep. Prog. Phys..

[34] Angelakis, D. G. (ed.) *Quantum Simulations with Photons and Polaritons*. Quantum Science and Technology (Springer, 2017).

[35] Rachel S, Laflorencie N, Song HF, Le Hur K (2012). Detecting quantum critical points using bipartite fluctuations. Phys. Rev. Lett..

[36] Koch J (2007). Charge-insensitive qubit design derived from the cooper pair box. Phys. Rev. A.

[37] Rozenberg MJ (1997). Integer-filling metal-insulator transitions in the degenerate hubbard model. Phys. Rev. B.

