# Author Correction: Nucleation of superfluid-light domains in a quenched dynamics

**DOI:** 10.1038/s41598-018-32564-2

**Published:** 2018-10-17

**Authors:** Joaquín Figueroa, José Rogan, Juan Alejandro Valdivia, Miguel Kiwi, Guillermo Romero, Felipe Torres

**Affiliations:** 1 Departamento de Física, Facultad de Ciencias, Universidad de Chile, Casilla 653, Santiago, 7800024 Chile; 2 Center for the Development of Nanoscience and Nanotechnology, 9170124 Estación Central Santiago, Chile; 3 Departamento de Física, Universidad de Santiago de Chile (USACH), Avenida Ecuador 3493, 9170124 Santiago, Chile

Correction to: *Scientific Reports* 10.1038/s41598-018-30789-9, published online 24 August 2018

This Article contains typographical errors in the Acknowledgements section.

“This work was supported by the Fondo Nacional de Investigaciones Científicas y Tecnológicas (FONDECYT, Chile) under grants No. 1150806 (FT), No. 1160639 (MK,JR), 1150718 (JAV), 11596590659 (GR), Grants-FA9550-16-1-0122 (FT,MK) and FA9550-18-1-0438 CEDENNA through the “Financiamiento Basal para Centros Científicos y Tecnológicos de Excelencia-FB0807” (FT, JR, MK and JAV).”

should read:

“This work was supported by the Fondo Nacional de Investigaciones Científicas y Tecnológicas (FONDECYT, Chile) under grants No. 1150806 (FT), No. 1160639 (MK,JR), 1150718 (JAV), 1150653 (GR), Grants-FA9550-16-1-0122 (FT,MK) and FA9550-18-1-0438 CEDENNA through the “Financiamiento Basal para Centros Científicos y Tecnológicos de Excelencia-FB0807” (FT, JR, MK and JAV).”

