# Supplementary material for: Nucleation of superfluid-light domains in a quenched dynamics
Source: Sci Rep. 2018 Aug 24;8:12766. doi: 10.1038/s41598-018-30789-9 (PMC6109166; doi:10.1038/s41598-018-30789-9)
Supplement: Supplementary file 1 — Supplementary Information [file 41598_2018_30789_MOESM1_ESM.pdf]

Supplementary Material: Nucleation of superfluid-light domains in a quenched dynamics

Joaquín Figueroa[1,2], José Rogan[1,2], Juan Alejandro Valdivia[1,2], Miguel Kiwi[1,2], Guillermo Romero[3], and Felipe Torres[1,2,†]

[1]Departamento de Física, Facultad de Ciencias, Universidad de Chile, Casilla 653, Santiago, Chile 7800024
[2]Center for the Development of Nanoscience and Nanotechnology 9170124, Estación Central, Santiago, Chile
[3]Departamento de Física, Universidad de Santiago de Chile (USACH), Avenida Ecuador 3493, 9170124, Santiago, Chile
[†]felestorres@gmail.com

Integration time

In the definition of the order parameter, namely, using the variance of the polariton number or the bipartite fluctuations of the polariton number, we integrate fluctuations up to a time $T = 1/J$, which is a characteristic time scale where the hopping strength dominates the dynamics, thus producing a delocalized wave function over different lattice points.

However, for longer simulation times, there are no significant differences in the behavior of the studied order parameter. We have carried out numerical simulations for the dimer, and for a linear array of three coupled resonators; the results are shown in Fig. (1). We see that the order parameter for different integration times exhibits the same qualitative behavior. Also, for times $t < 1/J$, not shown here, the studied order parameter almost vanishes and we do not see the crossover of the Mott-insulator to superfluid.

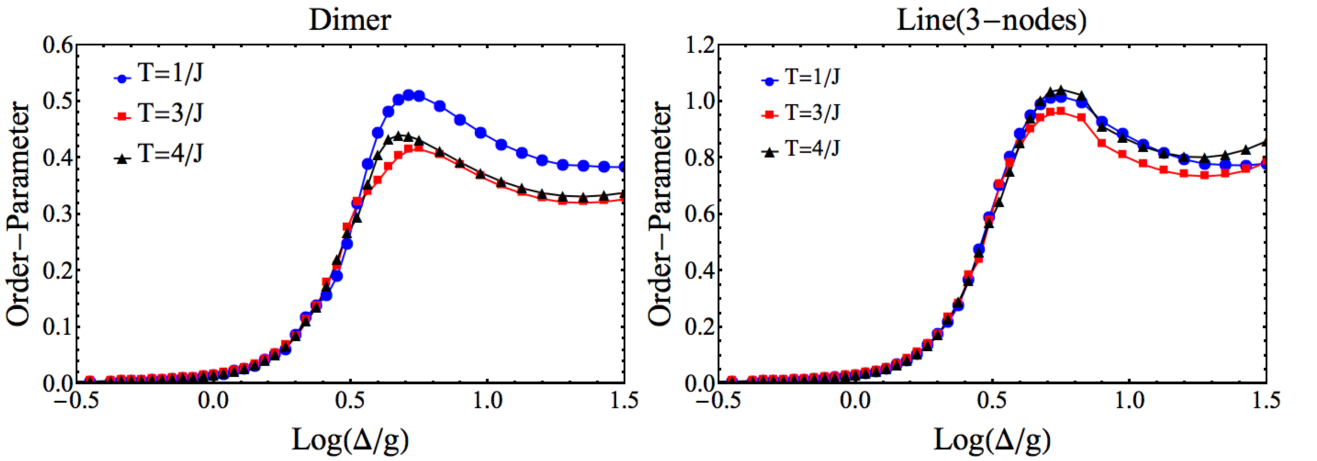

Figure 1. (color-online) Order parameter as a function of the detunig for different integration times ($T = 1/J$,$3/J$, and $4/J$). As the size of the array is increased there is no significant change in the standard deviation of the superfluid phase.

Describing Simulated First Order Phase Transition using Bipartite Fluctuations

The polariton number is conserved in our system since it does not exchange particles with the outside. Hence for both detuning $\Delta = 0$ and $\Delta \gg g$, where g stands for the light-matter coupling strength, the corresponding (simulated) Mott and superfluid states are described by Fock states. This way, if one considers the standard order parameter studied in mean-field approximation, that is, the average value of the annihilation operator per site, it will always be zero and will not capture any crossover from Mott insulator to superfluid. This is why we choose to study the simulated phase transition using the onsite variance of the polariton number. However, we demonstrate that the

first-order like-behavior is universal, in the sense that it does not depend on the choice of the order parameter. Indeed, if we use the bipartite fluctuations of subsystems we find that this approach also provides a correct description of the Mott to superfluid phase transition. Certainly, the dispersion of the polariton number on a given partition $M-th$ can be assessed by the variance of this subsystem, which can be obtained from the two-point correlation function $C_{i,j} = \langle n_i n_j \rangle - \langle n_i \rangle \langle n_j \rangle$, using the parameter $\sum_{i,j \in M} C_{ij}$, where n_i denotes the polariton occupation number at the i$-th$ site[1, 2].

We have performed numerical calculations for bipartite fluctuations in our finite arrays. Thus we can demonstrate that a simulated first order phase transition appears regardless of the choice of the order parameter; see left-panel of Fig. (2). As shown in the right-panel of the Fig. (2), using the bipartite fluctuations approach, the averaged standard deviation also displays a linear dependence on the connectivity of the partition.

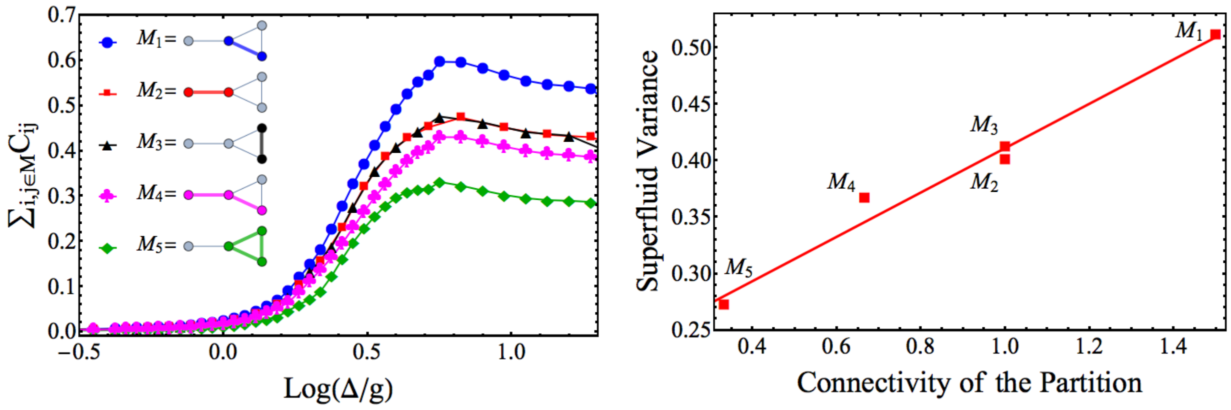

Figure 2. (color-online) (left) Bipartite fluctuations of polariton occupation number belonging to the M-th partition as a function of detunning. (right) Standard deviation of the superfluid phase as a function of the connectivity of the M-th partition.

Where the connectivity of the partition has been defined as the ratio between the external connectivity (total number of links between one node in the partition and the external nodes) of each node and the total number of nodes. The connectivity of the partition are summarized in Table 1. These results demonstrated the linear dependence of the order between the connectivity and the order parameter, in both case, when the order parameter is identified as the standard deviation or as the variance of the bipartite fluctuations.

Partition	External connectivity	Number of Nodes	Connectivity of the partition
M_1	2+1	2	3/2
M_2	2	2	2/2
M_3	2	2	2/2
M_4	2	3	2/3
M_5	1	3	1/3

Table 1. Summary of the connectivity of each partition corresponding to the array of the inset of Fig. (2-left). The variance of the superfluid phase depends linearly on the connectivity of the partition, as shown in Fig. (2-right)

Bose-Hubbard model

When we investigate the order parameter in the Bose-Hubbard model, in the framework of the grand canonical ensemble, it is apparent that it exhibits a similar behavior to the Jaynes-Cummings model, as shown in Figs. 3

The above results have been obtained for the Bose-Hubbard model in the grand canonical ensemble

$$H_{\rm BH} = -\mu \sum_j b_j^\dagger b_j + U \sum_j b_j^\dagger b_j^\dagger b_j b_j - J \sum_j (b_j^\dagger b_{j+1} + b_j b_{j+1}^\dagger),$$

and for a set of CRAs from two to eight interconnecting resonators, as shown in b) are considered. In order to reproduce an analogous quench dynamics, as compared with the Jaynes-Cummings-Hubbard model, we have adopted

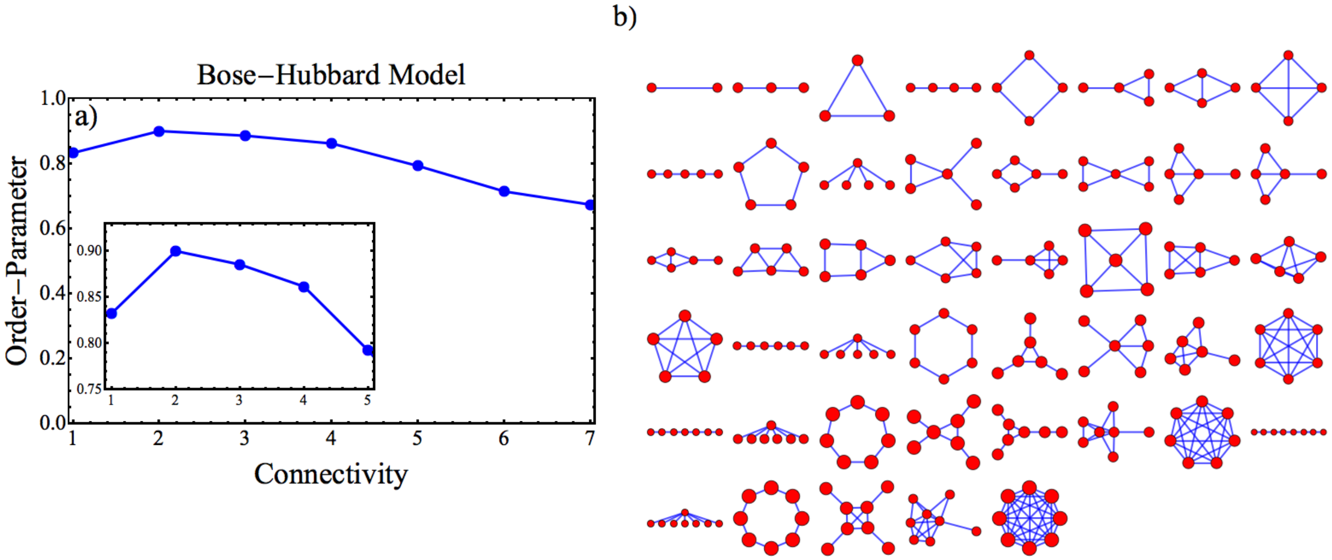

Figure 3. (color-online) a) Standard deviation of the bipartite fluctuations of the polariton occupation number as a function of connectivity in the Bose-Hubbard model. b) Set of CRAs for connectivities ranging from 2 to 7.

the parameters $\mu = 10^{-6}$, $J = 10^2\mu$, and U in the interval $[10^2 J, 10^{-2} J]$. As the chemical potential is not zero, and $U > \mu$, the lowest energy state per site in the Mott regime $U/J \gg 1$ is the Fock state $|1\rangle$. Thus, for each simulation our initial state is $|\psi_0\rangle = \bigotimes_{i=1}^{L} |1\rangle_i$.

References

1. S. Rachel, N. Laflorencie, H. Francis Song, and K. Le Hur, *Phys. Rev. Lett.* **108**, 116401 (2012), URL https://link.aps.org/doi/10.1103/PhysRevLett.108.116401.
2. D. Rossini, R. Fazio, and G. Santoro, *EPL* **83**, 47011 (2008), URL http://iopscience.iop.org/article/10.1209/0295-5075/83/47011?pageTitle=IOPscience.
